# Economic impact of clinical decision support interventions based on electronic health records

**DOI:** 10.1186/s12913-020-05688-3

**Published:** 2020-09-15

**Authors:** Daniel Lewkowicz, Attila Wohlbrandt, Erwin Boettinger

**Affiliations:** 1 Digital Health Center, Hasso Plattner Institute, University of Potsdam, Prof.-Dr.-Helmert-Str. 2-3, 14482 Potsdam, Germany; 2 Hasso Plattner Institute for Digital Health at Mount Sinai, Icahn School of Medicine at Mount Sinai, New York, NY 10029 USA

**Keywords:** Economic evaluation, Electronic health record, Clinical decision support, Behavioral economics

## Abstract

**Background:**

Unnecessary healthcare utilization, non-adherence to current clinical guidelines, or insufficient personalized care are perpetual challenges and remain potential major cost-drivers for healthcare systems around the world. Implementing decision support systems into clinical care is promised to improve quality of care and thereby yield substantial effects on reducing healthcare expenditure. In this article, we evaluate the economic impact of clinical decision support (CDS) interventions based on electronic health records (EHR).

**Methods:**

We searched for studies published after 2014 using MEDLINE, CENTRAL, WEB OF SCIENCE, EBSCO, and TUFTS CEA registry databases that encompass an economic evaluation or consider cost outcome measures of EHR based CDS interventions. Thereupon, we identified best practice application areas and categorized the investigated interventions according to an existing taxonomy of front-end CDS tools.

**Results and discussion:**

Twenty-seven studies are investigated in this review. Of those, twenty-two studies indicate a reduction of healthcare expenditure after implementing an EHR based CDS system, especially towards prevalent application areas, such as unnecessary laboratory testing, duplicate order entry, efficient transfusion practice, or reduction of antibiotic prescriptions. On the contrary, order facilitators and undiscovered malfunctions revealed to be threats and could lead to new cost drivers in healthcare. While high upfront and maintenance costs of CDS systems are a worldwide implementation barrier, most studies do not consider implementation cost. Finally, four included economic evaluation studies report mixed monetary outcome results and thus highlight the importance of further high-quality economic evaluations for these CDS systems.

**Conclusion:**

Current research studies lack consideration of comparative cost-outcome metrics as well as detailed cost components in their analyses. Nonetheless, the positive economic impact of EHR based CDS interventions is highly promising, especially with regard to reducing waste in healthcare.

## Contributions to the literature

- A recent economic analysis of implemented clinical decision support (CDS) interventions based on electronic health records (EHR) is presented based on previous reviews.
- Different EHR based CDS tools are prioritized and weighted regarding their economic benefit. This study provides policymakers, clinic managers, and other healthcare providers, who intend to implement similar health information technology, with a better understanding of valuable interventions and their application areas.
- The small number of model-based economic evaluations and the studies’ heterogeneity are regarded as indicators that information about costs and benefits is not extensively reported in the scientific literature.

## Background

As stated in the 2017 OECD health report, the annual average growth rate in per capita health expenditure continued to increase by 1.7% in Germany and 2.1% in the US in real terms since 2009 [1]. Healthcare expenditure per capita was estimated to be $5551 in Germany but was yet outspent by the United States, with almost 80% higher spending per capita [1]. The latest OECD Health Statistics 2019 report reconfirms these numbers on rising healthcare expenditure, and yet reveals an increase of spending per capita to $5986 in Germany and $10,586 in the US, which is equal to 11.2% and 16.9% of total GDP, respectively [2].

Unnecessary healthcare utilization, non-adherence to current clinical guidelines, or insufficient personalized care are perpetual challenges and remain potential major cost-drivers for healthcare systems around the world [3, 4]. For instance, a recent review estimated the annual cost of waste in the US healthcare system between $760 billion and $935 billion, which accounts for 25% of total healthcare spending [3]. Furthermore, Shrank et al. [3] approximated that $191 billion to $282 billion could be saved annually with the use of systematic interventions that address the reduction of waste in healthcare.

The benefits of electronic health records (EHR) culminate in the integration of computerized provider order entry (CPOE) systems and real-time, point of care clinical decision support (CDS) interventions. Introducing decision support systems into clinical care is promised to improve quality of care and thereby yield substantial effects on reducing healthcare expenditure [5]. In addition, the growing field of behavioral economics explores how different interventions, such as nudges or best-practice-alerts (BPA), influence and improve clinical decision making through various applicable concepts [6, 7].

The goal of this study is to explore the economic impact of EHR based CDS interventions and to identify a coherent best practice approach for these clinical interventions from a cost outcome perspective. Finally, we seek to examine cost-saving or cost-effective application areas for different medical risk factors.

## Methods

### Search strategy

We conducted a systematic literature review to identify the current research progress regarding the economic impact and benefits of clinical decision support interventions based on EHRs. Following the preferred reporting items for systematic reviews and meta-analyses (PRISMA) statement [8], we searched English-language literature indexed in the following databases: (1) PubMed, (2) Cochrane Central Register of Controlled Trials (CENTRAL) in the Cochrane Library, (3) Web of Science, (4) EBSCO Business Source Complete, and (5) CEA Registry Tufts Medical Center Library. Additionally, we screened the reference lists of all included studies for eligibility. The literature screening process was completed on January 10th, 2020.

To achieve high sensitivity and precision, we developed each search query based on three main pillars: (a) economic outcome, (b) electronic health record, and (c) clinical decision support. These main terms are then further extended with specific terminology and synonyms using Boolean operators to complete the search strategy. We used MeSH terms for the search in PubMed (1) and comparable search terms in databases (2)–(4). For the basic CEA registry search (5), only basic key search terms were used. A detailed summary of the developed search queries is listed in an additional file (see Additional file 1).

### Inclusion criteria

We included all trials in which a monetary economic outcome of an implemented EHR based CDS system is reported. Thus, we considered all analyses of inpatient or ambulatory financial data measures as well as trial-based modelling predictions. We also included all kinds of model perspectives, i.e., societal, health insurance, health systems, or user-centered perspective, to identify the complete economic dimension of an EHR based CDS intervention. We summarized the detailed inclusion and exclusion criteria of this systematic economic review in Table 1.

**Table 1:** Inclusion and exclusion criteria

	Inclusion criteria	Exclusion criteria
Type of decision support intervention	Any real-time and near real-time (point-of-care) computerized clinical decision intervention based on an EHR	− Decision support via e-mail, telephone contact, expert training or workshop, non-computerized education materials, or other behavioral economics interventions, such as accountable justification, i.e., free text entry, or peer comparison via e-mail − Retrospectively generated EHR based CDS alerts, e.g., for retrospective comparison or estimation − Basic CPOE without any decision stewardship − Cost or price display in order to facilitate cost-consciousness − BPA for EHR based patient recruitment for clinical trials − CDS for transitional care to improve post-discharge utilization and discharge management, i.e., process management − CDS usage for resource management, e.g., nurse staffing − EHR based CDS usage support through pay4performance incentives
Economic outcome	Monetary outcome data reported through quantitative cost-calculations or estimated through clinical trial-based modelling techniques	Other economic outcome measures, e.g., length of stay, amount of emergency department visits or primary care consultations

A pre-search showed that prior systematic reviews adequately elaborate the timeframe until 2014, and we, therefore, include only studies published from 2014 to 2020. During our search process, we found that the number of studies meeting the inclusion criteria increased tremendously in the past years, partially overlapping the present research question [9–11]. The most recent review by Jacob et al. [9] examined the cost and economic benefits of CDS systems restricted to cardiovascular disease prevention. However, the authors were unable to conclude whether these interventions were cost-beneficial or cost-effective. Moja et al. [10] reviewed randomized controlled trials (RCTs) that examined the effectiveness of EHR based CDS systems with regard to mortality, morbidity, and economic outcomes. The authors report that EHR based CDS interventions resulted in only small differences in cost and health service utilization.

### Front-end CDS interventions

Wright et al. [12] developed a taxonomy of front-end CDS interventions available to EHR users, which we adopt into this study. In contrast to back-end system capabilities, the authors defined front-end CDS tools as “the intervention types available to end-users created using specific clinical knowledge bases and application logic [12].” Their taxonomy consists of 53 designed CDS front-end tools, i.e., interventions, that were further categorized into six categories [12]:

1. Medication dosing support
2. Order facilitators
3. Point-of-care alert or reminders
4. Relevant information display
5. Expert systems
6. Workflow support

We utilize this taxonomy to analyze application areas of significant cost-savings. For this, we prioritize and weight different EHR based CDS tools based upon their economic benefit. This approach can give policymakers, clinic managers, and other healthcare providers a better understanding of valuable EHR based CDS interventions and their application areas to implement similar health information technology.

## Results

**Fig. 1:**
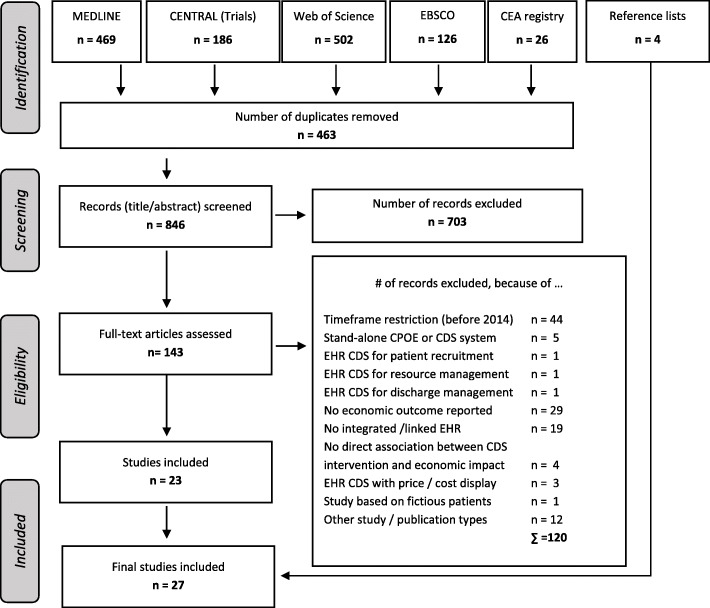
Flow-diagram of the search process (*N* = 1309 publications screened)

We screened in total 1309 publications, of which 27 studies meet our inclusion criteria for this economic review [5, 13–38]. The process of our literature search and the reasons for excluding several studies is provided within the PRISMA flow-diagram in Fig. 1. An overview of the characteristics of the included studies is listed in Table 2.

**Table 2:** Characteristics of included studies (*n* = 27)

Category	Number of studies (% of total, rounded)
Country	
United States	24 (89%) [5, 13–18, 20, 22, 23, 25–38]
Canada	3 (11%) [19, 21, 24]
Year published	
2019	6 (22%) [13–18]
2018	6 (22%) [5, 19–23]
2017	5 (19%) [24–28]
2016	2 (7%) [29, 30]
2015	3 (11%) [31, 33, 34]
2014	5 (19%) [32, 35–38]
Study design	
Cluster randomized trial	4 (15%) [5, 19, 26, 33]
Cross-sectional	1 (4%) [28]
Retrospective	9 (33%) [15, 17, 18, 20, 21, 27, 32, 36, 38]
Quasi-experimental	5 (19%) [14, 16, 22, 24, 35]
Comparative	1 (4%) [31]
Observational	1 (4%) [23]
Pre-post-intervention	6 (22%) [13, 25, 29, 30, 34, 37]
Setting	
Inpatient	14 (51%) [14, 16, 17, 20, 22–25, 27, 31, 32, 34, 36, 38]
Outpatient	8 (30%) [5, 13, 15, 19, 26, 30, 33, 34]
Inpatient & outpatient	4 (15%) [21, 28, 29, 37]
Emergency department	1 (4%) [18]
Type of economic evaluation	
Basic cost calculation	23 (85%) [13–25, 27–32, 34, 36–38]
Model approach	4 (15%) [5, 26, 33, 35]

Generally, 22 studies (81%) [5, 13–16, 18, 20–25, 28–32, 34–38] out of the included 27 studies report cost savings after implementing an EHR based CDS intervention. Four studies (15%) [17, 26, 27, 33] report a rise in cost expenditure. The remaining study (4%) [19] did not detect significant differences in cost outcomes. In the majority of included studies the main cost outcome measures were related to laboratory test cost [15–17, 20, 21, 25, 28, 29, 31, 32, 38].

### Exploration of different front-end CDS intervention categories

According to the taxonomy by Wright et al. [12], we identified 10 (37%) studies [5, 13, 15, 20, 22, 23, 26, 36–38] which explored EHR based CDS interventions based on *point-of-care alerts or reminders* (category 3). Three interventions (11%) [17, 27, 34] were *order facilitators* (category 2). *Medication dosing support, relevant information display,* and *expert systems* (categories 1, 4, and 5) were each reported only once from an economic perspective (11%) [18, 19, 24]. In eight studies (30%) [14, 16, 25, 28, 30, 31, 33, 35], interventions from two different categories were explored in combination. Moreover, we found three studies (11%) [21, 29, 32] in which the option to place a certain order or test, e.g., a laboratory test, was removed from the EHR CPOE system or the clinician’s laboratory ordering preference list. These restrictive frond-end CDS intervention types were not yet mentioned in the predefined categories by Wright et al. [12]. Thus, we extend their taxonomy by a new category:

7. Restriction of choice [39]

The removal of an order option ultimately resulted in fewer laboratory tests and reduced healthcare expenditure in all studies [14, 28, 31]. Finally, we identified two different types of implemented hard-stops [40]: an interruptive alert [30] and a restrictive hard-stop [14]. An interruptive alert requires a clicking response from the physician before being able to move forward. A restrictive hard-stop prevents the physician from ordering a test, e.g., by directing them to call the laboratory director. We grouped studies regarding the interruptive alert intervention to category 3 and the restrictive hard-stop intervention to category 7.

### Economic impact for prevalent application areas

In Table 3, we summarized our findings and created an overview of application areas and cost outcome measures in relation to the applied CDS intervention types. The included studies show a high heterogeneity with regard to different types of reported cost outcomes and different intervention durations. Due to this complexity, it was not possible to conduct a subgroup analysis regarding the economic impact of each CDS-front end category. A detailed evidence synthesis of all included 27 studies and a brief description of their intervention types, their application area, and the resulting economic impact are provided in an additional file (see Additional file 2).

**Table 3:** Application areas and cost outcome measures in relation to CDS intervention categories 1.-7

Study	Size^a^	Application area	CDS intervention period *(in month)*	Change in cost outcome per year *(in US$, if not other stated)* ^b^	
				per patient	per activated alert
**1. Medication (dosing) support**					
Tamblyn [19]	Medium	Reduce out-of-pocket cost for patients with uncomplicated hypertension	60	*no difference*^d^	
**2. Order facilitator**					
Bolles [17]	Small	Inappropriate test ordering for specialized HIV laboratory testing	6	+$102 to +$670	
Schnaus [27]	Large	The order “complete blood count without differential” unintentionally changed to “complete blood count with differential”	23 days	+$8	
Shaha [34]	Small	CDS order sets for managing new-onset stroke patients	6	-$1742 to -$4280	
**3. Point of care alerts or reminders**					
Gong [5]	Medium	Inappropriate antibiotic prescribing for acute respiratory infection	18	-$0.16^c^	
Chen D [13]	Large	Reduce unnecessary imaging studies in patients with low back pain	12	-$30	
Chin [15]	Large	Decrease routine testing for 25(OH) vitamin D levels	12	-$65	
Bejjanki [20]	Large	Reduce 17 frequently used duplicate laboratory tests	17	n/a ^f^	
Chen JR [22]	Small	Directing the physician to order penicillin allergy testing for patients receiving aztreonam	9	-$678	
Heekin [23]	Large	Adherence to 18 different Choosing Wisely (CW) alerts	36	-$944	
Sharifi [26]	Small	Clinical childhood obesity intervention	12	+$11^c^	
Goodnough [36]	Large	Reduce overutilization in blood transfusion procedure	36	-$308	
Razavi [37]	Small	Reduce unnecessary waste in transfusion practice and blood use of cardiothoracic surgeons	12	-$82	
Bridges [38]	Small	Reduce unnecessary acute hepatitis profile laboratory tests	3	-$20	
**4. Relevant information display**					
Fertel [18]	Small	Reduce the amount of frequent or high emergency department utilizers	24	-$24,672	
**5. Expert systems**					
Nault [24]	Large	Antimicrobial stewardship that facilitates the post-prescription review process	36	- CAD $10	
**6. Workflow support**					
none	–	–	–	–	
**7. Restriction of choice**					
MacMillan [21]	Large	Reduce unnecessary frequent red blood cell folate tests	43	- CAD $5	
Konger [29]	Large	Define order frequency rules and reduce duplicate tests	24	n/a ^g^	
Procop (b) [32]	Large	Reduce unnecessary, same day duplicate orders	24	-$8	
Studies with combined multiple CDS intervention categories					
**1. Medication (dosing) support & 3. Point of care alerts or reminders**					
Stenner [30]	Large	ePrescribing tool for therapeutic interchange prescribing	18	-$17	
Forrester [35]	Medium	CPOE CDS vs. paper-based prescribing in reducing medication errors and adverse drug events (ADE)	10	-$6^c^	
**2. Order facilitator & 3. Point of care alerts or reminders**					
Goetz [16]	Large	Decrease serum folate laboratory testing	12	-$29	
**2. Order facilitator & 6. Workflow support**					
Michaelidis [33]	Medium	Reduce inappropriate antibiotic prescribing for acute bronchitis	6	+$8^c^	
**2. Order facilitator & 7. Restriction of choice**					
Sadowski [25]	Medium	Reduce admission order sets, which allowed multiple routine tests to be ordered repetitively	2	-$55^e^	
**3. Point of care alerts or reminders & 7. Restriction of choice**					
Marcelin [14]	Large	Reduce inappropriate gastrointestinal pathogen panel testing	15	n/a ^h^	
Felcher [28]	Medium	Reduce unnecessary Vitamin D testing	6	-$157	
Procop (a) [31]	Medium	Unnecessary duplicate laboratory testing	12	Hard-Stop -$16.08Smart-Alert -$3.52	

^a^ Size is defined as the following:

Number of patients or encounters involved

0–999 small size

1000–10,000 medium-size

> 10,000 large size

If the patient count was not reported, we applied this range of criteria to the number of triggered alerts in total

^b^ All cost outcomes were scaled and calculated to the overall change in cost outcome per year and per patient or activated alert. Values (for > $1) are rounded to full integer numbers. Because of the predominantly short CDS intervention period time range, a discount factor is not used for calculation. The originally reported cost data is mentioned in an additional file (see Additional file 2) [41–45]

^c^ Cost estimation based on a model

^d^ No statistically significant differences between control and intervention group regarding out-of-pocket cost per patient

^e^ Estimated reduced cost per inpatient day per year after intervention 1

^f^ No information regarding the number of patients or alerts. Overall cost outcome per year: - $51,206

^g^ No information regarding the number of patients or alerts. Overall cost outcome per year: - $157,782

^h^ No information regarding the number of patients or alerts. Overall cost outcome per year: -$53,600

#### Application areas for cost-savings

We identified four primary application areas based on their investigated prevalence that resulted in cost-savings after EHR based CDS implementation. Firstly, two studies report on reducing unnecessary Vitamin D routine testing, which led to a decrease in laboratory test cost of $300,000 [15] and $1.4 mill. [28] per year.

Secondly, two studies addressed the economic outcome of reducing waste in transfusion practice and red blood cell usage [36, 37]. The acquisition product cost of red cell units was decreased with the help of EHR based CDS and resulted in cost savings of $4,821,000 after 3 years [36] and about $62,715 within 1 year [37] after implementation, respectively.

Thirdly, two cost-effectiveness analyses modeled the cost outcome of reducing antibiotic prescriptions for acute respiratory infection and acute bronchitis [5, 33]. Gong et al. [5] include a full accounting of costs into their Markov model, and explore that the implemented CDS intervention, called “suggested alternatives”, yielded more quality adjusted life years (QALYs) at a lower cost of $500,000 per 100,000 individuals over 30 years of implementation. Michaelidis et al. [33] report only a small increase in costs compared to a printed decision support system, i.e., posters. However, the latter mainly results from a cost difference between the direct costs of poster printing and computer programming.

Lastly, five studies [20, 29, 31, 32, 38] report on the potential to reduce duplicate orders, e.g., duplicate laboratory tests, using hard-stops [32] or order frequency rules [20]. Order frequency rules prevent ordering the same test within a specified timeframe. Reducing duplicate laboratory tests resulted in savings of $3395 after 3 months for a small patient size cohort [38], and up to $315,565 within 24 month for a large patient size cohort [29].

#### Application areas resulting in cost increase

We also identified risk areas, which possibly lead to a further increase in healthcare expenditure. One study found that specialized HIV laboratory testing cost increased by $14,000–$96,000 within 6 months after implementing a CPOE system with default settings [17]. Another study reports that an unplanned change of a pre-selected default order for ‘complete blood count’ to ‘complete blood count with differential’ led to an average cost increase of $293.11 per day [27]. Finally, the implementation of order sets as decision facilitators possibly entails adverse economic effects. One study found that only after uncoupling joint orders of Vitamin B12 and serum folate tests from predefined order sets, laboratory test cost decreased by about $26,719 per year [16]. Similarly, another study removed the option to order daily routine tests from automated admission order sets and found savings of $26,416 after 2 months [25].

### Cost-effectiveness-analysis models

In Table 4, we present an overview of studies that conducted a cost-effectiveness-analysis (CEA) of EHR-based CDS interventions and include various cost data as well as economic outcome measures. One such economic outcome measure is the incremental cost-effectiveness ratio (ICER), which depicts the incremental change in costs divided by the incremental change in health outcome or effect.

**Table 4:** Overview of cost data and cost outcome of model-based studies (*n* = 4)

Study	Model time horizon (years)	Choice of model	Implementation and maintenance costs	Total budget impact	ICER
Gong et al. [5]	30	Markov model	$1.91 base case for 100,000 individuals [preexisting EHR]	CDS intervention $17.32 mill. Control $17.82 mill.	$99.8 per QALY in base case scenario
Sharifi et al. [26]	10	Monte Carlo micro-simulation	$23,542 per PCP group [preexisting EHR]	CDS intervention +$239 mill.	$237 per BMI unit reduction
Michaelidis et al. [33]	5	Decision analytic tree	$18 base case - medical record programming [preexisting EHR]	CDS intervention $2802^a^ Control (usual care) $2768^a^	$51.51 per antibiotic prescription safely avoided
Forrester et al. [35]	5	Decision analytic tree	$1,773,000 5 years CPOE system cost	CDS CPOE system $25 mill. Control (paper system) $43mill.	$110 per ADE averted^b^

^a^Cumulative 5-year societal cost per five cases of acute bronchitis

^b^Documented only for the explored modelling scenario no. 2: The Everett Clinic achieved no reduction in paper chart pulls throughout the 5-year time horizon, to explore the effect of inefficiency from running a paper and electronic system in parallel

Cost-effectiveness analyses aim to reveal the trade-offs in resource-allocation decisions [46]. In this context, it is essential to investigate when and to what extend upfront and maintenance costs for an EHR based CDS system will be amortized by its benefits. The benefits can be measured in health outcomes, such as quality adjusted life years (QALYs), or the reduction of unnecessary healthcare utilization.

Generally, two studies report an increase in healthcare expenditure from a societal perspective [26, 33] while the other two report cost savings from a societal perspective and the medical group’s perspective [5, 35]. Notably, the measurement of effectiveness was single study-based estimates in all four studies.

Regarding the consideration of upfront implementation cost, Gong et al. [5] include only base case consolidated cost data of $1.91 for a cohort of 100.000 individuals based on expert opinions. Sharifi et al. [26] include intervention start-up cost for EHR modification of $2.7 mill. as well as other direct costs, such as professional care provider training. Michaelidis et al. [33] report implementation and maintenance cost data, which is physician education per hour and medical record and CDS programming per patient of $18 in the base case. Lastly, Forrester et al. [35] report CPOE CDS system cost as hardware, software, and maintenance costs starting from $373,000 in year one to $92,000 after 5 years, as well as personnel, $555,000 in year one, and indirect costs as 3% of the total cost. Interestingly, the indirect costs also include the HITECH Meaningful Use incentives in their model to simulate the financial incentives by the Centers for Medicare & Medicaid Services in the US [35, 47].

### Studies lack consideration of all cost components

Despite revealing significant potentials for cost-savings, we could not assess the quality of the included studies because of missing cost information or non-consideration of all relevant cost components. According to the Consolidated Health Economic Evaluation Reporting Standards (CHEERS) statement, most of the reported recommendations were not satisfied [48]. All 23 non-model studies (85%) only calculate the economic outcome based on financial data reported before and after intervention implementation. For instance, this results from the computation of price per healthcare resource utilization multiplied by the quantity of used healthcare resources or services. Thus, even though it was not intended in those studies, it is necessary to mention that only four of the included studies adhere to sound economic evaluations as recommended by CHEERS [5, 35].

The challenge of heterogeneity for the CEA is also aggravated by considering different cost outcomes. Two studies neither report an incremental cost effectiveness ratio (ICER) for a predefined threshold directly, nor do they include comparative metrics [33, 35]. Other standardized metrics, such as the return on investment or net present value, were also not examined in the included studies. Only one study reports on the net monetary benefit (NMB) of the intervention in relation to a predefined threshold [5, 49].

### Additional studies worth mentioning

Notably, five more studies [50–54] meet most of our inclusion criteria but were excluded due to various, although little, deviations. Three studies [50–52] report cost-savings after a bundle of information technology was implemented simultaneously, but the economic benefit could not solely be attributed to the EHR based CDS intervention. The fourth publication is an NHS health technology assessment (HTA) report [53]. In this HTA, an RCT was conducted in 79 general practices in the UK in which a multi-component intervention was installed using EHRs to reduce the number of antibiotic prescriptions for respiratory infections. The authors perform a basic cost-analysis focusing on the number of provider consultations as the cost of healthcare utilization. However, they found no difference in the cost outcome between the intervention and control period.

The last study worth mentioning compared retrospectively generated alerts by an advanced machine learning CDS system to alerts triggered through the home-grown EHR based CDS system [54]. The authors calculated the healthcare cost of potentially prevented adverse drug events and medication errors and found that the advanced machine learning CDS system gave 68.2% more alerts resulting in cost savings of $60.67 per alert. After extrapolating these results to a local patient population of 747,985, they estimated savings of $1,294,457 over 5 years [54].

## Discussion

Evaluating the economic impact of EHR-based CDS interventions and their potential to increase value in healthcare remains a significant challenge. Even though we found that 22 studies report cost savings, most of them do not include developing or maintaining costs. Therefore, we could not draw a sound correlation between vendor-purchased or home-grown systems’ costs to their economic benefit. Nonetheless, this study reveals several use cases with coherent CDS tools that have proven to be cost-saving and could be eligible for other healthcare providers, clinic managers, and researchers for implementation or further exploration.

With the majority of implemented CDS interventions based on point-of-care alerts, the question remains on how more algorithm-based expert systems and multiple interventions will have synergy effects on the economic impact. Considering the amount of alerts and the healthcare provider’s time expense, a process-cost analysis, such as the time-driven activity based costing approach, could be combined with the CEA to achieve a better understanding of the whole cost cycle as well as productivity effects for healthcare entities [55, 56]. Generally, cost outcome measures continue to require comparative metrics, for instance, as used by Mathias et al., the cost per useful alert [57]. In a simple model, the authors introduce this measure to analyze how different parameters affect the cost of implementing EHR based CDS alerts for genomic precision medicine [57]. However, for future economic evaluations of EHR based CDS interventions, a more specific approach for individual application areas or focus on medical risk factors is needed to draw meaningful conclusions from cost and outcome comparisons [9]. Moreover, decision-analytic modelling techniques, e.g., Markov models, enable the evaluation of multiple income and outcome parameters. They address downstream costs or savings that may result from the introduction of health information technology. These complex modelling approaches are necessary in order to consider various health outcomes resulting from EHR CDS systems, e.g., prevention of adverse events [5].

Another economic challenge to consider is CPOE systems with default lists or opt-out options of orderable tests as well as predefined order sets. The automation of orders through such order sets or joint-order options could ultimately lead to an increase in costs and a decrease of value [58, 59]. For example, the rate of unnecessary laboratory tests can increase when healthcare professionals tend to accept the whole order set rather than de-selecting single order items [17]. This can be explained by alert fatigue, which must not directly be related to the order set, in combination with the ‘button clicking syndrome’, which explains the inducement of moving along inattentively [17, 59]. Apart from the direct economic factors, other potential benefits of order sets and joint-order options, such as improved adherence to clinical guidelines or patient safety outcomes, are not sufficiently addressed by the included studies. This again highlights the importance of further profound health economic evaluations.

Finally, one study also reported an increase in costs after an unplanned change in the CDS system [27]. Such malfunctions or unintended errors, when caused by newly integrated health information technology, are referred to as ‘e-Iatrogenesis’ [60]. These may also lead to yet another cost-driver and possibly cause unpredictable economic damage [61, 62].

### Transferability for other countries

All included studies where based on cost data and trials from the United States or Canada. Consequently, current research progress on the economic potentials of EHR based CDS systems on rising healthcare expenditure in Europe and worldwide cannot be derived. We found recent studies that evaluate the cost-effectiveness of a stand-alone CPOE CDS system in the Netherlands [63], or compare the effectiveness of an EHR based CDS intervention in the US, UK, Republic of Korea, and Belgium [64]. Another RCT explored the effectiveness of an EHR based CDS intervention for patients with atrial fibrillation and a high risk of stroke in Sweden [65]. Nevertheless, we found no study that has evaluated the potential for increasing value in this present highly promising field of health information technology outside of North America.

However, this study reveals promising cost savings for already implemented health information technology. Even though implementation cost was not considered, on a long-term view, these results reveal the potential for cost-savings once implementation cost is amortized. Therefore, the sooner large health information technology systems will be implemented in other countries around the world and evaluated economically, the earlier cost-benefits and return on investments can be realized.

### Support from policymakers could accelerate economic benefits

Interestingly, Forrester et al. [35] include monetary incentives provided by the Meaningful Use Initiative in the US in their CEA. This financial support covered only a small percentage of total implementation cost in their developed model as incentive-eligible prescribers received $42,000 over 5 years. Nevertheless, this contributed to the investigated cost-effectiveness of an EHR based CDS intervention compared to paper-based prescriptions. Therefore, how policymakers worldwide intend to support EHR adoption and incentivize embedded CDS systems financially is a critical factor for the economic success of such systems. High upfront implementation cost constitute a significant burden for healthcare entities, especially for smaller to middle-sized practices and hospitals [66]. For instance, a systematic review of EHR embedded CDS systems for cardiovascular disease prevention derived the mean annual cost of development, implementation, and ongoing costs of operations to be $102 per patient and $6056 per practice for small practices, and $49 per patient and $35,201 per practice for medium-sized practices [9].

Finally, achieving a decrease in healthcare expenditure should never influence a patient’s quality of life or disease treatment in a negative way. Even though eliminating a laboratory order option from a CPOE system led to cost-savings, each patient’s value and health outcome is of the highest importance and should be individually assessed. Future economic evaluations of EHR based CDS systems should focus more on the potentials of health benefits that could be achieved, such as through reduced antibiotic prescriptions or reduced adverse drug events, rather than proving to have effectively reduced laboratory test cost. In the end, competing on shifting costs will not change anything about the primary goal of decision stewardship, and that is to increase value in healthcare [67].

### Limitations

There are some limitations to this study. Firstly, we only considered English language literature and, therefore, might not have included international publications in other languages that indeed report on the information technology progress made by other countries regarding the linkage of CDS systems to an existing EHR. Another limitation is the exclusion of EHR cost and price display interventions. This decision was based on another recent systematic review, which found that cost display interventions in EHR CPOE systems do not affect the efficiency and effectiveness domain of healthcare quality [68]. We also excluded other non-monetary impact measures, such as length of stay, which necessarily also refers to the economic impact of EHR based CDS implementations.

Overall, our findings might be biased since we included all types of studies as well as all kinds of monetary outcomes. Due to the lack of economic evaluations, included studies tend to declare high cost-savings but only consider little to no cost components regarding the implementation and maintenance of such a complex information system. In addition, authors might have been tempted to calculate cost-savings only when the implemented intervention proved effective. Finally, we found that some studies not necessarily mention the calculated economic outcome in the title or abstract of their publication. Therefore, we might have excluded studies that did not initially meet our inclusion criteria by following the PRISMA guidelines.

## Conclusion

Clinical decision support interventions based on electronic health records have an overall positive economic impact. Predominantly point-of-care alerts concerning unnecessary laboratory testing, efficient transfusion practice, or reduction of antibiotic prescription emerged as application areas with already promising potential for high-cost savings. Nonetheless, most studies lack consideration of coherent cost components as well as comparative metrics. Therefore, the economic dimension of EHR based CDS interventions needs to be further explored. High-quality cost-effectiveness or cost-utility analyses, which include more extensive cost data and consider different economic perspectives, are needed to draw a sound conclusion. Finally, introducing personalized health services based on peoples’ electronic health records is yet another promising research field with high potential for further increasing value in healthcare and should receive more attention in future research.

## Supplementary information

**Additional file 1.** Search strategy and developed search terms for different databases.

**Additional file 2. **Evidence synthesis and summary of included studies (*n* = 27).

## Data Availability

Not applicable.

## Abbreviations

ADE: Adverse drug events
BPA: Best-practice-alerts
CDS: Clinical decision support
CEA: Cost-effectiveness-analysis
CHEERS: Consolidated Health Economic Evaluation Reporting Standards
CPOE: Computerized provider order entry
CW: Choosing Wisely
EHR: Electronic health record
HTA: Health technology assessment
ICER: Incremental cost-effectiveness ratio
NMB: Net monetary benefit
QALY: Quality adjusted life years
RCT: Randomized controlled trial
PRISMA: Preferred Reporting Items for Systematic Reviews and Meta Analyses
