# Supplementary material for: Economic impact of clinical decision support interventions based on electronic health records
Source: BMC Health Serv Res. 2020 Sep 15;20:871. doi: 10.1186/s12913-020-05688-3 (PMC7491136; doi:10.1186/s12913-020-05688-3)
Supplement: Supplementary file 1 — Additional file 1. Search strategy and developed search terms for different databases. [file 12913_2020_5688_MOESM1_ESM.docx]

**Search strategy and developed search terms for different databases**

1. Search query for: **PubMed**

(Economics [MeSH] OR ("economic evaluation" OR "cost-effectiveness" OR "cost-utility" OR "cost-benefit" OR "economic intervention" OR cost OR price OR "cost saving" OR “return on investment” OR “cost minimization” OR Decision Support Systems, Clinical/economics* OR Economics, Behavioral*)) AND (Decision Support Systems, Clinical [MeSH] OR ("clinical decision support" OR “decision support” OR "computerized decision support" OR CDS OR "best practice" OR “alert system” OR “computerized provider order entry” OR CPOE OR “computer assisted” OR “Computerized Physician Order entry”)) AND (Electronic health record [MeSH] OR ("electronic health record" OR "health record" OR "electronic patient record" OR "patient record" OR "electronic medical record" OR "medical record" OR EHR OR EMR))

1. Search query for: **Cochrane Library Central (Trials), Web of Science, EBSCO**

(Economic* OR ("economic evaluation" OR "cost-effectiveness" OR "cost-utility" OR "cost-benefit" OR "economic intervention" OR cost OR price OR "cost saving" OR “return on investment” OR “cost minimization” OR Economics, Behavioral)) AND (Decision Support Systems, Clinical OR ("clinical decision support" OR “decision support” OR "computerized decision support" OR CDS OR "best practice" OR “alert system” OR “computerized provider order entry” OR CPOE OR “computer assisted” OR “Computerized Physician Order entry”) ) AND (Electronic health record OR ("electronic health record" OR "health record" OR "electronic patient record" OR "patient record" OR "electronic medical record" OR "medical record" OR EHR OR EMR))

1. Basic key-word search: **CEA Registry**

(1) Electronic health record (2) Electronic medical record (3) Electronic patient record (4) Clinical decision support system (5) Intervention (6) Computerized provider / physician order entry
