# Supplementary material for: Economic impact of clinical decision support interventions based on electronic health records
Source: BMC Health Serv Res. 2020 Sep 15;20:871. doi: 10.1186/s12913-020-05688-3 (PMC7491136; doi:10.1186/s12913-020-05688-3)
Supplement: Supplementary file 2 — Additional file 2. Evidence synthesis and summary of included studies (n = 27). [file 12913_2020_5688_MOESM2_ESM.docx]

**Evidence synthesis and summary of included studies (n=27)**

| **Economic cost impact** | ↓ | | | ↓ | ↓ | ↓ | ↑ | ↓ | 🡨🡪 ³ |
| --- | --- | --- | --- | --- | --- | --- | --- | --- | --- |
| **Monetary outcome**  **(in US $, if not stated)** | -$1,872,000 / year | | | -$67,000 „hard stop”-$168,000 cost savings of additional GIPP encounters prevented / 15 months | - $300,000 / year | - $26,719.56 / year | between  + $14,000 -$92,000  / 6 months | - $3,855.3 average monthly per care plan | CAD$252.6 (control)  CAD$261.5  (intervention) / patient year |
| **Implementation or maintenance cost included** | No | | | No | No | No | No | No | No |
| **Type of cost outcome reported** | Cost of lumbar spine MRI | | | Cost of GIPP testing + cost savings based on the number of encounters GI pathogen orders that were opened and avoided | Laboratory test cost | Laboratory test cost | Laboratory test cost (using different cost databases) | Technical direct and indirect cost related to providing patient care | Out-of-pocket-costs |
| **Intervention period**  (total in month) | 12 | | | 15 | 12 | 12 | 6 | 24 | 60 |
| **Application area** | Reduce unnecessary imaging studies in patients with non -specific low back pain within the first six weeks | | | Reduce inappropriate gastrointestinal pathogen panel (GIPP) testing | Decrease routine testing for 25(OH) vitamin D levels | Decrease serum folate laboratory testing | Reduce inappropriate test ordering for specialized HIV laboratory testing | Reduce the amount of frequent or high ED utilizers | Reduce out-of-pocket costs for newly or currently treated patients with uncomplicated hypertension |
| **Related front-end CDS category** | 3 | | | 3 & 7 | 3 | 2 & 3 | 2 | 4 | 1 |
| **Type of CDS intervention approach** | Pop-up BPA on the frequency of lumbar imaging in patients with acute low back pain | | | Providing guidance and a “hard stop”alert to avoid duplicate testing or tests for patients hospitalized >72h  + silent best practice advisory when users opened the GIPP order panel | BPA explained low utility of testing, when an order for 25(OH) vitamin D was placed | Advisory warning on the folate laboratory order + uncoupling of the default joint order set “B12 and serum folate testing” | CPOE with default settings (facilitates the “button clicking syndrome”) | BPA to prompt individual care plans (ICP), when providers entered the patient’s chart | (1) Alert with alternate drug choices and out-of-pocket costs  (2) Pop-up suggests more cost-effective therapies |
| **Size** **¹** | Medium | | | Medium | Large | Medium | Small | Small | Medium |
| **Study** | Chen D et al.[13] | | | Marcelin et al.[14] | Chin et al.[15] | Goetz et al.[16] | Bolles et al.[17] | Fertel et al.[18] | Tamblyn et al.[19] |
| **Economic cost impact** | ↓ | | | ↓ | ↓ | ↓ | ↓ | ↓ | ↓ |
| **Monetary outcome**  **(in US $, if not stated)** | -$ 500,000 /  30 years and 100,000 individuals | | | - $72,543 /  17 months | -CAD$280,145  / 43 months | - $673.73 /  per patient | -$944  / patient (median  encounter cost $12,940) | -CAD $350,000  / year | - $26,416.37  / 2 months |
| **Implementation or maintenance cost included** | Yes | | | No | No | No | No | No | No |
| **Type of cost outcome reported** | Societal cost | | | Laboratory test cost | Laboratory test cost | Antibiotic cost | Total direct costs | Antimicrobial spending costs | Laboratory test cost |
| **Intervention period**  (total in month) | 18 | | | 17 | 43 | 9 | 36 | 36 | 2 |
| **Application area** | Inappropriate antibiotic prescribing for acute respiratory infection (ARI) | | | Reduce 17 elected and frequently used duplicate laboratory tests | Reduce frequent red blood cell (RBC) folate tests | Directing the physician to order penicillin allergy testing for patients receiving aztreonam | Adherence to the 18 highest volume Choosing Wisely CW alerts | Antimicrobial stewardship that facilitates the post-prescription review process | Reduce admission order sets, which allowed multiple routine tests to be ordered repetitively |
| **Related front-end CDS category** | 3 | | | 3 | 7 | 3 | 3 | 5 | 2 & 7 |
| **Type of CDS**  **intervention approach** | CDS pop-up followed by a list of suggested alternatives in lieu of antibiotic prescribing | | | BPA “pop up” window informed, if a recent identical order already existed within predefined window periods | The option to order the lab test was removed from physicians' EHR CPOE | CDS to advice penicillin allergy skin testing | CDS intervention pops up when initiating a potentially inappropriate order | CDS alerts for potentially inappropriate prescriptions using validated algorithms | Removal of option to order daily routine laboratory tests from automated admission order set |
| **Size** **¹** | Medium | | | Large | Large | Small | Large | Large | Medium |
| **Study** | Gong et al.[5]  *based on*  Meeker et al.[64] | | | Bejjanki et al. [20] | MacMillan et al.[21] | Chen JR et al.[22] | Heekin et al.[23] | Nault et al.[24] | Sadowski et al.[25] |
| **Economic cost impact** | ↑ | (↑)² | | | ↓ | ↓ | ↓ | ↓ | ↑ |
| **Monetary outcome**  **(in US $, if not stated)** | + $175mill. /  10 year, population reach 2mill. | + $293.11 / average daily cost | | | - $1,4 mill. /  year | -$ 315,565 /  24 months | -$203,239  / quarter | Hard-Stop:  -$94,225  Smart-Alert:  -$45,681  / 12 months | +$34  5-year societal cost per five cases of acute bronchitis |
| **Implementation or maintenance cost included** | Yes | No | | | No | No | No | No | Yes |
| **Type of cost outcome reported** | Societal costs | Complete blood count ordering cost | | | Laboratory test cost | Laboratory test cost | Medication prescribing cost | Laboratory test cost | Societal costs |
| **Intervention period**  (total in month) | 12  BMI obtained at initial- and 1-year follow-up visits | 23 days | | | 6 | 24 | 18 | 12 | 6 |
| **Application area** | Clinical childhood obesity intervention | The order “complete blood count without differential” within the CDS system unintentionally changed to “complete blood count with differential | | | Reduce Vitamin D testing | Define order frequency rules and reduce specific (duplicate) tests or test panels for certain patient cohorts | ePrescribing Tool for therapeutic interchange prescribing | Unnecessary duplicate laboratory testing | Inappropriate antibiotic prescribing for acute bronchitis |
| **Related front-end CDS category** | 3 | 2 | | | 3 & 7 | 7 | 1 & 3 | 3 & 7 | 2 & 6 |
| **Type of CDS**  **intervention approach** | CDS alerts pediatrician of BMI >95^th^ percentile and displaying links for further information | CDS CPOE default setting: An unintentional change of a pre-selected order (the test order is highlighted and referenced first within a list of orders) | | | (1) Introducing a new vitamin D screening guideline, (2) "Hard stop" alert requiring acknowledgement, (3) Vitamin D testing was removed from the laboratory ordering preference list | CDS intervention declines certain order requests, if the order exceeds the predefined rule for order frequency | "Hard stop" pop-up alert to inform that an interchange is recommended | Comparison between two CDS tools (1) Hard-Stop: required telephone contact  (2) Smart Alert: possible to bypass the alert directly | BPA (1) to provide educational brochure to patient (2) displays structured documenting template (3) create intended order sets |
| **Size** **¹** | Small | Large | | | Medium | Large | Large | Medium | Medium |
| **Study** | Sharifi et al.[26]  *based on*  Taveras et al.[65] | Schnaus et al.[27] | | | Felcher et al.[28] | Konger et al.[29] | Stenner et al.[30] | Procop et al. (a)[31] | Michaelidis et al.[33]  *based on*  Gonzales et al*.*[66]  *commented by*  Black[67] |
| **Economic cost impact** | ↓ | | ↓ | | ↓ | ↓ | ↓ | ↓ |  |
| **Monetary outcome**  **(in US $, if not stated)** | Between  - $230,000 and $565,000  / 6 months | | - $18 mill.  / 10.000  Monte Carlo simulations | | -$1,620,000  / year | -$62,715.90  / year | -$183,586  / 24 months | -$3,395  / 3 months |  |
| **Implementation or maintenance cost included** | No | | Yes | | No | No | No | No |  |
| **Type of cost outcome reported** | Pre-Post intervention cost comparison | | Healthcare provider cost | | Acquisition product costs | Transfusion cost | Laboratory test cost | Laboratory test cost |  |
| **Intervention period**  (total in month) | 6 | | 10 | | 36 | 12 | 24 | 3 |  |
| **Application area** | CDS-driven  order sets for managing new-onset stroke patients | | CPOE CDS vs. paper-based prescribing in reducing medication errors and adverse drug events | | Reduce overutilized blood transfusion procedure.  BPA for transfusions when hemoglobin level > 7 g/dL | Reduce waste in transfusion practice and blood use of cardiothoracic surgeons | Reduce unnecessary and same day duplicate orders | Reduce the number  and cost of duplicated Acute Hepatitis Profile (AHP) laboratory tests |  |
| **Related front-end**  **CDS category** | 2 | | 1 & 3 | | 3 | 3 | 7 | 3 |  |
| **Type of CDS**  **intervention approach** | Order sets alerts. reflecting 13 patient care and documentation elements | | Alert with basic dosing guidance, duplicate therapy checks, and pediatric dosing calculations | | BPA at time of order entry - "interruptive alert" with consensus guidelines and link to relevant literature | CPOE CDS provides transfusion guidelines and patient hemodynamic data. Requires CDS specific form to complete. | A hard-stop alert that a same-day duplicate test was being ordered (i.e., a pop-up box) + display of most recent results and final list of 1,259 tests. | CDS pop up screen  informed, if the ordered test was pending results, or scheduled in the future. |  |
| **Size** **¹** | Small | | Medium | | Large | Small | Large | Small |  |
| **Study** | Shaha et al.[34] | | Forrester et al.[35]  *based on*  Devine et al.[68] | | Goodnough et al.[36] | Razavi et al.[37] | Procop et al. (b)[32] | Bridges et al.[38] |  |

**Denotation**

¹Size / population is defined as the following:

Number of patients and/or encounters involved

0-999 small size

1,000-10,000 medium size

>10,000 large size

If patient count was not reported, we applied this range of criteria to the amount of alerts / interventions

²Comparative study: Expenditure increase resulted from the unintentional change within the EHR CDS system

³No statistically significant differences between control and intervention out-of-pocket costs per patient

**General:** economic cost impact:

**↑ Increase in healthcare expenditure**

= Total cost outcome was higher in the EHR CDS intervention group than in the control group or pre-implementation

**↓** **Decrease in healthcare expenditure**

= Total cost outcome was lower in the intervention group than in the control group or pre-implementation.
